# Correlation analysis between molecular subtypes and Nottingham Prognostic Index in breast cancer

**DOI:** 10.18632/oncotarget.18242

**Published:** 2017-05-27

**Authors:** Hongchao Zhen, Liuting Yang, Li Li, Junxian Yu, Lei Zhao, Yingying Li, Qin Li

**Affiliations:** ^1^ Department of Oncology, Beijing Friendship Hospital, Capital Medical University, Beijing, 100050, China; ^2^ Department of Biochemistry and Molecular Biology, Basic Medical College, Shanxi Medical University, Taiyuan, 030001, China; ^3^ Department of Pharmacy, Beijing Friendship Hospital, Capital Medical University, Beijing, 100050, China; ^4^ Department of Pathology and Pathophysiology, Basic Medical College, Capital Medical University, Beijing, 100069, China

**Keywords:** molecular subtypes, Nottingham Prognostic Index, breast cancer, prognosis, correlation analysis

## Abstract

Molecular subtypes and Nottingham Prognostic Index (NPI) are both prognostic models for breast cancer patients. We evaluated the association between molecular subtypes and NPI in 1042 breast cancer patients. The molecular subtypes indicating poorer prognosis were positively correlated to higher NPI (*r* = 0.138, *P =* 0.001). ER positive expression and PR high expression were positively correlated with NPI (*r* = 0.142, *P =* 0.001; *r* = 0.139, *P =* 0.001; respectively) and negatively correlated with histological grade (*r* = −0.233, *P* < 0.001; *r* = −0.176, *P* < 0.001; respectively). Ki67 status was negatively correlated with NPI and positively correlated with histological grade (*r* = −0.120, *P* =0.004; *r* = 0.197, *P* < 0.001; respectively). The percentages of cases with NPI score 2.00–3.40 were higher in the luminar A, ER+, PR high expression and Ki67 low expression group, and the percentages of cases with NPI > 5.40 were higher in the HER2 overexpression subtype, basal-like subtype, ER-, PR low/negative expression, and Ki67 high expression groups. The excellent consistence was observed between histological grade and molecular subtypes, ER, PR, Ki67. The difference of histological grade between the HER2 positive and negative group was statistically significant. In conclusion, there was closely association between molecular subtypes and NPI in breast cancer. For further comparing the prognostic significance of molecular subtypes and NPI, survival analyses should be performed on the same population in a large-scale prospective study.

## INTRODUCTION

Breast cancer is the most common malignant tumor in women around the world, comprising 25% of all cancer cases in women [1]. Because of its high morbidity, breast cancer seriously affects women’s health and life quality. At present, surgical resection is the main treatment strategy for patients with breast cancer. Adjuvant chemotherapy, endocrine therapy, radiation therapy, targeted therapy and other comprehensive treatments have also significantly improved disease free survival and overall survival. However, postoperative recurrence and metastasis are still big problems plagued the clinicians and patients. Thus, there is an urgent need to establish effective models for evaluating prognosis of breast cancer.

The Nottingham Prognostic Index (NPI) established in 1982 is a widely used clinicopathological score system for primary breast cancer prognostication [2]. The NPI combines nodal status, tumor size and histological grade in a simple formula. It stratifies breast cancer patients into three prognostic groups: good, moderate and poor [3]. The NPI could guide individualized therapeutic decision for breast cancer by providing risk stratification. Its advantages of prognosis stratification have been confirmed by various studies, and it has been widely used in clinical practice [4–6]. However, the NPI does not address biological and molecular characteristics of breast cancer.

In recent years, prognostic significance of gene expression has become the research hotspot. For example, the 21-gene recurrence score, 70-gene signature and TP53 mutation-correlated genes were verified to have prognostic value in different breast cancer populations [7–9]. However, the expensive cost of the multi-gene assays and the lack of verifiable trials prohibit them for clinical application in many countries [10]. So the experts propose utilizing routinely pathological parameters to replace gene detection in clinical practice. In 2013, the St Gallen Consensus Conference and ESMO Clinical Practice Guidelines recommended surrogate definitions of intrinsic molecular subtypes of breast cancer [11]. According to the expression of estrogen receptor (ER), progesterone receptor (PR), human epidermal growth factor receptor 2

(HER2) expression/amplification and Ki67, breast cancer is classified into four molecular subtypes including luminal A, luminal B, HER2 overexpression and the basal-like subtype. Endocrine therapy is generally effective for patients with the luminal A subtype, and this type shows the best outcome [12]. Luminal B subtype, determined by the status of Ki67 and HER2, appear obvious distinction from luminal A [13, 14]. Luminal B shows poorer prognosis compared with luminal A [15, 16]. Whereas, HER2 overexpression subtype and basal-like subtype are associated with a higher risk of recurrence and present the worse outcome [17–20].

In this study, we evaluated the correlation and inherent links between the molecular subtypes and NPI in breast cancer, with the aim to further understand and find the best prediction model in breast cancer. We also analyze the relationships among the sub-factors in two models.

## RESULTS

### Correlation analysis of different variables between molecular subtypes and NPI

Spearman correlation analysis was used to calculate the correlation of different variables in molecular subtypes and NPI. The analysis confirmed a significant positive correlation between molecular subtypes and NPI. The molecular subtypes indicating poorer prognosis were positively correlated to higher NPI (*r* = 0.138, *P* = 0.001). ER positive expression and PR high expression were positively correlated with NPI (r = 0.142, *P* = 0.001; r = 0.139, *P* = 0.001; respectively) and negatively correlated with histological grade (r = −0.233, *P* < 0.001; r = −0.176, *P* < 0.001; respectively). Ki67 status was negatively correlated with NPI and positively correlated with histological grade (r = −0.120, *P* =0.004; r = 0.197, *P* < 0.001; respectively). HER2 status had no significant association with NPI and its components including tumor size, lymph node staging and histological grade. (Table 1)

**Table 1:** Correlation analysis of different variables between molecular subtypes and Nottingham Prognostic Index (NPI)

		NPI score	Grade	Tumor	Node
**Molecular subtypes**	*r*	0.138	−0.212	0.005	−0.026
	*P* value	0.001	0.000	0.891	0.540
**ER**	*r*	0.142	−0.233	0.027	−0.041
	*P* value	0.001	0.000	0.471	0.330
**PR**	*r*	0.139	−0.176	0.056	−0.024
	*P* value	0.001	0.000	0.132	0.567
**HER2**	*r*	0.000	0.063	0.023	0.003
	*P* value	0.999	0.141	0.553	0.944
**Ki67**	*r*	−0.120	0.197	−0.059	−0.012
	*P* value	0.004	0.000	0.119	0.779

### Cross analysis between molecular subtypes and NPI

**Figure 1:**
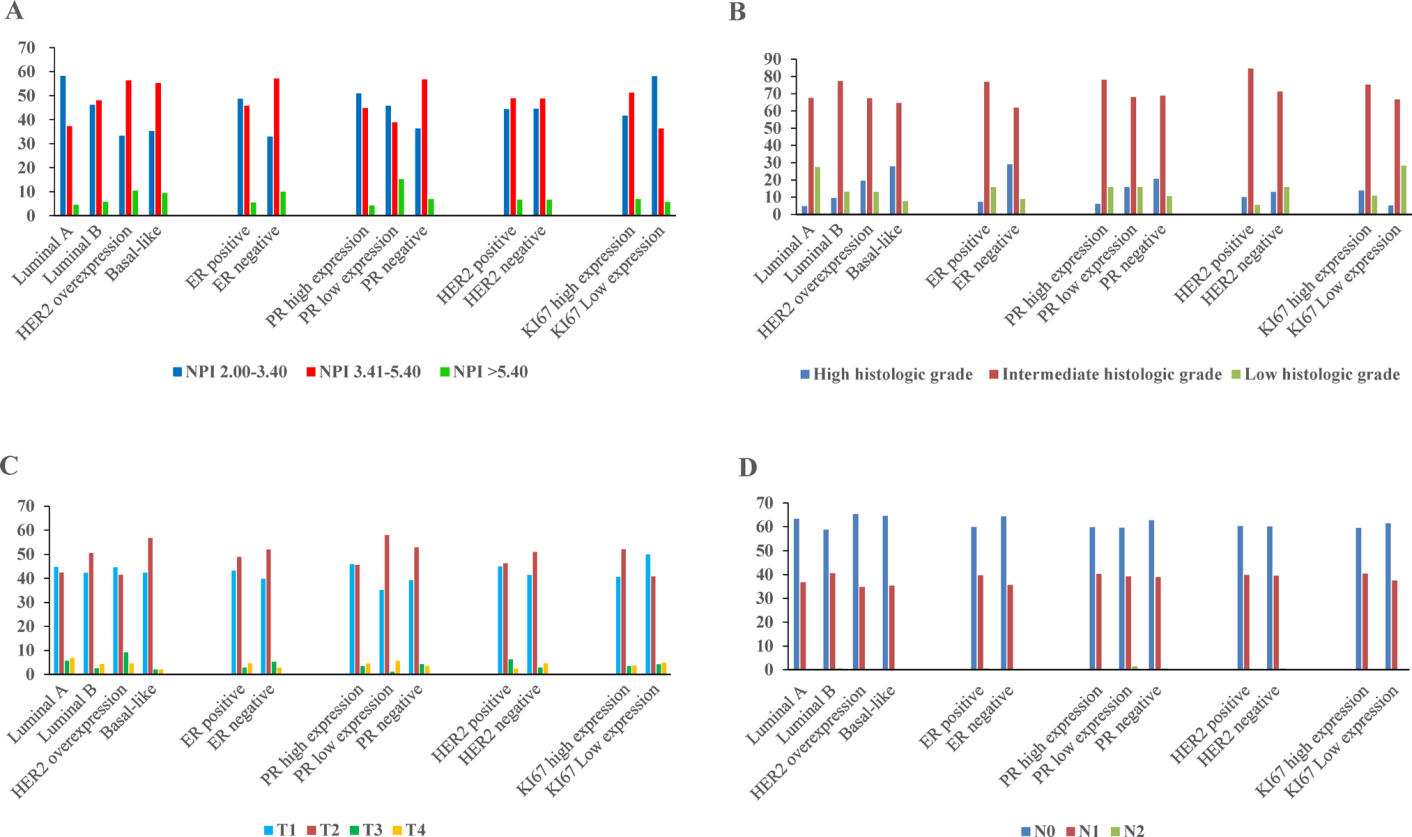
The distribution of NPI variables in molecular subtypes and its sub-factors (**A**) The proportion of NPI scores in molecular subtypes and its sub-factors. (**B**) The proportion of histological grades in molecular subtypes and its sub-factors. (**C**) The proportion of tumor sizes in molecular subtypes and its sub-factors. (**D**) The proportion of lymph nodes in molecular subtypes and its sub-factors.

The distribution percentages of NPI scores and its components in the molecular subtypes are shown in Table 2 and Figure 1. The percentages were luminal A > luminal B > HER2 overexpression or basal-like in the NPI 2.00–3.40 group, whereas, the percentages were luminal A < luminal B < HER2 overexpression or basal-like in the NPI > 5.40 group. The percentages were luminal A < luminal B < HER2 overexpression < basal-like in the high histological grade group, whereas, the percentages were luminal A > luminal B > HER2 overexpression > basal-like in the low histological grade group. The differences of the histological grade in the four molecular subtypes were statistically significant (*P* < 0.001). However, there are no significant differences of tumor size or lymph nodes in the four molecular subtypes. Luminal B subtype included HER2 negative and HER2 positive subgroups, and there was no difference of NPI score between two subgroups (*P* = 0.305). However, the difference of NPI score between HER2+/ER+ and HER2+/ER- subtypes was statistically significant (*P* = 0.038).

**Table 2:** Cross analysis between molecular subtypes and Nottingham Prognostic Index (NPI)

	Luminal A *n* (%)	Luminal B *n* (%)	HER2 overexpression *n* (%)	Basal-like *n* (%)	*X*^2^	*P*
**NPI score**					12.051	0.061
2.00–3.40	39 (58.2)	183 (46.2)	16 (33.3)	30 (35.3)		
3.41–5.40	25 (37.3)	190 (48.0)	27 (56.3)	47 (55.3)		
> 5.40	3 (4.5)	23 (5.8)	5 (10.4)	8 (9.4)		
**Grade**					35.240	0.000
High histological grade	3 (4.8)	36 (9.5)	9 (19.6)	22 (27.8)		
Intermediate histological grade	42 (67.7)	293 (77.3)	31 (67.4)	51 (64.6)		
Low histological grade	17 (27.5)	50 (13.2)	6 (13.0)	6 (7.6)		
**Tumor size**					15.409	0.220
T1	39 (44.8)	198 (42.4)	29 (44.6)	38 (39.1)		
T2	37 (42.6)	237 (50.6)	27 (41.5)	55 (56.7)		
T3	5 (5.7)	12 (2.7)	6 (9.3)	2 (2.1)		
T4	6 (6.9)	20 (4.3)	3 (4.6)	2 (2.1)		
**Lymph nodes**					2.432	0.876
N0	38 (63.3)	220 (58.8)	30 (65.2)	51 (64.6)		
N1	22 (36.7)	152 (40.7)	16 (34.8)	28 (35.4)		
N2	0 (0.0)	2 (0.5)	0 (0.0)	0 (0.0)		

### Cross analysis of ER status and NPI

The percentage of cases with NPI score 2.00–3.40 in the ER+ group was significantly higher than that in the ER- group, the percentage of cases with NPI score > 5.40 in the ER- group was nearly 2.0 fold than that in ER+ group, and the difference of NPI score between the two groups was statistically significant (*P* = 0.002). The opposite trends were observed regarding histological grade and ER status. There were no significant differences of tumor size or lymph nodes between ER+ and ER- groups. (Table 3 and Figure 1)

**Table 3:** Relationship between ER status and Nottingham Prognostic Index (NPI)

	ER positive *n* (%)	ER negative *n* (%)	***X***^2^	*P*
**NPI score**			12.081	0.002
2.00–3.40	221 (48.7)	46 (32.9)		
3.41–5.40	208 (45.8)	80 (57.1)		
> 5.40	25 (5.5)	14 (10.0)		
**Grade**			44.816	0.000
High histological grade	32 (7.4)	39 (29.1)		
Intermediate histological grade	332 (76.9)	83 (61.9)		
Low histological grade	68 (15.7)	12 (9.0)		
**Tumor size**			4.019	0.403
T1	238 (43.4)	68 (39.8)		
T2	269 (48.9)	89 (52.0)		
T3	16 (2.9)	9 (5.3)		
T4	26 (4.8)	5 (2.9)		
**Lymph nodes**			1.405	0.495
N0	256 (59.8)	85 (64.4)		
N1	170 (39.7)	47 (35.6)		
N2	2 (0.5)	0 (0.0)		

### Cross analysis of PR status and NPI

The relationship between NPI scores and PR status was shown in Table 4 and Figure 1. These results were similar to the relationship between NPI scores and ER status. The percentage of cases with NPI score 2.00–3.40 in the PR high expression group was higher compared with that of PR low expression and negative group. The percentage of cases with NPI score > 5.40 in the PR low expression and negative groups was significant higher than that in the PR high expression group, and the difference of NPI score among the three groups was statistically significant (*P* < 0.001). The percentage of cases with high histological grade in the PR high expression group was significant lower than that in the PR low expression and negative group. However, these significant trends were not observed in tumor size and lymph nodes staging among different PR groups.

**Table 4:** Relationship between PR status and Nottingham Prognostic Index (NPI)

	PR high expression *n* (%)	PR low expression *n* (%)	PR negative *n* (%)	*X*^2^	*P*
**NPI score**				22.319	0.000
2.00–3.40	157 (51.0)	33 (45.8)	78 (36.3)		
3.41–5.40	138 (44.8)	28 (38.9)	122 (56.7)		
> 5.40	13 (4.2)	11 (15.3)	15 (7.0)		
**Grade**				25.441	0.000
High histological grade	18 (6.1)	11 (15.9)	41 (20.6)		
Intermediate histological grade	232 (78.1)	47 (68.2)	137 (68.8)		
Low histological grade	47 (15.8)	11 (15.9)	21 (10.6)		
**Tumor size**				9.326	0.316
T1	172 (46.1)	31 (35.2)	101 (39.3)		
T2	171 (45.8)	51 (58.0)	136 (52.9)		
T3	13 (3.5)	1 (1.1)	11 (4.3)		
T4	17 (4.6)	5 (5.7)	9 (3.5)		
**Lymph nodes**				3.718	0.446
N0	169 (59.7)	44 (59.5)	126 (62.7)		
N1	114 (40.3)	29 (39.2)	74 (36.8)		
N2	0 (0)	1 (1.3)	1 (0.5)		

### Cross analysis of HER2 status and NPI

In the retrospective study, our results showed that HER2 status was closely related to the histological grade. The percentage of cases with low histological grade in the HER2+ group was significant lower than that in the HER2- group. However, there were no significant distribution differences of NPI, tumor size and lymph nodes staging between HER2+ and HER2- groups. (Table 5 and Figure 1)

**Table 5:** Relationship between HER2 status and Nottingham Prognostic Index (NPI)

	HER2 positive *n* (%)	HER2 negative *n* (%)	*X*^2^	*P*
**NPI score**			0.001	0.999
2.00–3.40	60 (44.4)	198 (44.5)		
3.41–5.40	66 (48.9)	217 (48.8)		
> 5.40	9 (6.7)	30 (6.7)		
**Grade**			11.351	0.003
High histological grade	13 (10.0)	55 (13.0)		
Intermediate histological grade	110 (84.6)	300 (71.1)		
Low histological grade	7 (5.4)	67 (15.9)		
**Tumor size**			6.055	0.109
T1	72 (45.0)	217 (41.4)		
T2	74 (46.2)	267 (51.1)		
T3	10 (6.3)	15 (2.9)		
T4	4 (2.5)	24 (4.6)		
**Lymph nodes**			0.588	0.745
N0	74 (60.2)	252 (60.0)		
N1	49 (39.8)	166 (39.5)		
N2	0 (0.0)	2 (0.5)		

### Cross analysis of Ki67 status and NPI

We found that the percentage of cases with NPI score 2.00–3.40 was elevated in the Ki67 low expression group compared with that of the Ki67 high expression group, the percentage of cases with NPI score 3.41–5.40 was lower in the Ki67 low expression group than that in the Ki67 high expression. The difference of NPI scores and histological grade between the two groups were statistically significant (*P* = 0.009; *P* < 0.001). However, there was no significant difference in the tumor size or lymph nodes staging between Ki67 high expression and low expression groups. (Table 6 and Figure 1)

**Table 6:** Relationship between Ki67 status and Nottingham Prognostic Index (NPI)

	Ki67 high expression *n* (%)	Ki67 low expression *n* (%)	*X*^2^	*P*
**NPI score**			9.402	0.009
2.00–3.40	196 (41.7)	61 (58.1)		
3.41–5.40	241 (51.3)	38 (36.2)		
> 5.40	33 (7.0)	6 (5.7)		
**Grade**			23.322	0.000
High histological grade	62 (13.8)	5 (5.1)		
Intermediate histological grade	338 (75.3)	66 (66.7)		
Low histological grade	49 (10.9)	28 (28.3)		
**Tumor size**			5.723	0.126
T1	223 (40.6)	71 (50.1)		
T2	286 (52.1)	58 (40.8)		
T3	19 (3.5)	6 (4.2)		
T4	21 (3.8)	7 (4.9)		
**Lymph nodes**			1.848	0.397
N0	266 (59.4)	54 (61.4)		
N1	181 (40.4)	33 (37.5)		
N2	1 (0.2)	1 (1.1)		

### Clinicopathological data for the entire patient cohorts

All clinicopathplogical data could be obtained from Supplementary Tables 1–3.

## DISCUSSION

Breast cancer is classified into four molecular subtypes according to the expression of ER, PR, HER2 and Ki67. Molecular subtypes as new prognostic indicators have been received more and more attention. In these molecular subtypes, Luminal A subtype shows the best outcome, whereas, HER2 overexpression and basal-like subtypes present the poorer outcome [15–20]. Breast cancer patients with the same clinical prognostic profile may have markedly different outcomes, which are concrete manifestations of distinct molecular biology behavior [21]. Traditional clinicopathological parameters including positive margin, vascular tumor invasion, histological grade, lymph node staging and tumor size have been verified as independent risk factors for recurrence [22–27]. NPI combines the number of involved lymph nodes, tumor size and histological grade to determine prognosis, it is a well-established clinicopathological score system which offers comprehensive prognostic information than single marker [28, 29]. The markers involving in molecular subtypes and NPI are completely different, however, both are used to guide treatment and predict prognosis. Whether the novelty molecular subtypes could completely substitute the traditional clinicopathological prognostic markers is a question worthy of consideration, and whether traditional NPI provide more detailed stratification in every molecular subtypes is another question worthy of in-depth research. However, the correlation and inherent link between the molecular subtypes and NPI have rarely been reported. The in-depth understanding of the association between the two classifications will help us to choose the precise prognostic markers in clinical practice.

In this study, we conducted a comprehensive analysis on the relationship between the molecular subtypes and NPI in breast cancer. The overall analysis confirmed that the molecular subtypes were significantly correlated with the traditional NPI score, the molecular subtypes indicating poorer prognosis were positively correlated to higher NPI score. ER+ and PR+ as good prognostic indicators positively correlated with NPI scores and negatively correlated with histological grade. Ki67 as proliferation indicator negatively correlated with NPI scores and positively correlated with pathological grade. Higher percentages of NPI score 2.00–3.40 were seen in the luminar A, ER+, PR high expression and Ki67 low expression group, and higher percentages of NPI > 5.40 were seen in the ER-, PR low/negative expression and Ki67 high expression groups. There had excellent consistence between NPI, histological grade and molecular subtypes, ER, PR and Ki67. The study of Kurshumliu F demonstrated that good prognosis markers, such as ER positive expression, PR positive expression and Ki-67 low expression, were seen with higher frequency in good and moderate NPI groups [4]. This was consistent with our findings.

Specimens that display a basal-like cell phenotype features are also called, in routine practice, as triple negative breast cancer (TNBC). It represents an easily recognizable breast cancer group with aggressive behavior [30]. TNBC are shown to have an attenuated relationship between tumor size, nodal status, and survival. It is currently accepted that TNBC is more prone to haematogenous metastasis rather than lymph node metastasis [31]. Since lymph nodal is a major component for NPI calculation, there are concerns about the reliability of using NPI as a tool for TNBC prognostication. However, Dent R reported a higher prevalence of lymph node metastasis in TNBC [32], and Albergaria A had demonstrated that TNBC disseminated to axillary lymph nodes as frequently as other subtypes, it had the ability to predict the survival of TNBC, and NPI was a truthful prognostic tool in TNBC [6]. In our study, the proportion of N1+N2 in basal-like subtype was almost the same as that in other subtypes.

Tumor size is another major component of NPI. Generally speaking, it is independent risk factor for recurrence [27]. In our study, the percents of T2 were significant higher than percentages of T1 in basal-like subtype group, ER negative group, PR negative/low expression group. However, the same trend was not observed in HER2 over expression group and HER2 positive group. One study reported that there was an increased tumor size for the ER-/PR-/HER2+ and ER-/PR-/HER2- subtypes, however, Foulkes WD proposed that basal-like breast cancers and HER2 positive breast cancer were inherently aggressive and were likely to early metastasize, tumor size might not be related to the prognosis [33, 34]. Cheang MC put forward that the special type (ER−/PR−/HER2−/cytokeratin 5+ and/or EGFR+) of basal-like breast cancers might have cancer stem-like properties and the strong tendency to metastasize early [35]. These maybe lead to some conflict results about tumor size and outcome in the breast cancers.

In conclusion, there is closely association between molecular subtypes and NPI in breast cancer. For further comparing the prognostic significance of molecular subtypes and NPI, survival analyses should be performed on the same population in a large-scale prospective study. Moreover, it is necessary to evaluate the prognostic significance of NPI in different molecular subgroups or the additional value of intrinsic molecular classification to different NPI score. Different prognostic models have their one-sidedness, and the comprehensive analysis will better guide the clinician to judge the prognosis.

## MATERIALS AND METHODS

### Study population

During the period between January 2008 and December 2012, a total of 1,042 patients with operable breast cancer were enrolled in the study, and average age ± standard deviation was 55.56 ± 12.37 years (range, 22 to 92 years). All patients were either from the Department of Breast Surgery or Department of Oncology. Biopsies or surgical resection specimens were pathologically examined and histologically confirmed. Complete pathological records were available, and the following details were recorded: tumor size (T), the number of positive lymph nodes (N), histological tumor grade (G), histopathological type, ER, PR, Ki67 and HER2 status. All patients were not treated with radiation before operation. The patients with the history of other malignant disease and recurrent malignancies were excluded. Patients who had neoadjuvant chemotherapy were not excluded because the NPI retained its prognostic value after this form of treatment [36]. The research was reviewed and approved by the Ethics Committee of Beijing Friendship Hospital in China. All procedures performed in the study involving human participants were in accordance with the ethical standards of the Beijing Friendship Hospital, Capital Medical University and/or national research committee and with the 1964 Helsinki declaration and its later amendments or comparable ethical standards. Before collecting human samples, all participants signed informed consent forms according to our institutional guidelines.

### Traditional histological characteristics

T, N, G and histopathological type were collected and classified according to the American Joint Committee on Cancer TNM Staging System for Breast Cancer (National Comprehensive Cancer Network Guidelines Version 2.2016 for Breast Cancer). G analysis was centrally performed on whole sections according to the recommendations of Nottingham combined with histological grade (Elston-Ellis modification of Scarff-Bloom-Richardson grading system), also known as the Nottingham Grading System.

### Scoring for immunohistochemistry (IHC)

ER, PR and Ki67 status were determined by immunohistochemical staining. Qualitative scoring of ER and PR was performed using ASCO/CAP criteria, and an IHC scoring was used as follows: +, total score 3–4; ++, total score 5–6; +++, total score 7–8. Tumors were considered HER2 positive if scored 3+ by immunohistochemical staining or 2+ by immunohistochemical staining and also HER2 amplified (ratio > 2.0) on the basis of fluorescence *in situ* hybridization. In the absence of positive fluorescence *in situ* hybridization data, tumors scored 2+ by immunohistochemical staining were considered negative for HER2.

### Definitions for molecular subtypes of breast cancer

Four molecular subtypes (luminal A, luminal B, HER2-overexpression and basal-like) were classified. Table 7 was surrogate definitions of molecular subtypes of breast cancer according to the 2013 St Gallen Consensus Conference and ESMO Clinical Practice Guidelines.

**Table 7:** Surrogate definitions of molecular subtypes of breast cancer

Molecular Subtypes	Luminal A	Luminal B	HER2 overexpression	Basal-like
histopathologic surrogate definition	• ER-positive	HER2-negative	HER2-positive (non-luminal)	Triple-negative (ductal)
	• HER2-negative	• ER-positive	• HER2-positive	• ER and PR absent
	• Ki67 low*	• HER2-negative	• ER and PR absent	• HER2-negative
	• PR high**	• and either		
		• Ki67 high** or		
		• PR low*		
		HER2-positive		
		• ER-positive		
		• HER2-positive		
		• any Ki67		
		• any PR		

Notes: *The cut-off point between high and low values for Ki-67 varies among laboratories. **Suggested cut-off values for both PR and Ki-67 are 20%. Abbreviations: ER, oestrogen receptor; PR, progesterone receptor; HER2, human epidermal growth factor receptor 2.

### NPI scoring

Whenever possible, NPI was calculated for each patient using the following equation: NPI = (0.2 × S) + G + N [37]. In this formula, S is the tumor size in cm, N is the number of involved lymph nodes (>4 = 3, 4–1 = 2, 0 = 1), and G is the degree of malignancy of the tumor (degree 3 = 3, degree 2 = 2, degree 1 = 1). All patients were assigned into one of the three different prognosis groups: good, 2.00–3.40; moderate, 3.41–5.40; poor prognostic, > 5.40 [3].

### Statistical analysis

All analysis was performed using the SPSS 13.0 software (SPSS Inc., Chicago, Illinois, USA). The categorical variables were described using numbers and percentage. The correlation analysis of the categorical variables was analyzed by Spearman correlation analysis. The relationships between molecular subtypes and NPI were evaluated using Chi-square test. A two sided *P* value less than 0.05 was considered to be statistically significant.

## SUPPLEMENTARY MATERIALS TABLES



## Abbreviations

NPI: Nottingham Prognostic Index
ER: estrogen receptor
PR: progesterone receptor
HER2: human epidermal growth factor receptor 2
TNBC: triple negative breast cancers
