# Supplementary material for: Correlation analysis between molecular subtypes and Nottingham Prognostic Index in breast cancer
Source: Oncotarget. 2017 May 27;8(43):74096–105. doi: 10.18632/oncotarget.18242 (PMC5650325; doi:10.18632/oncotarget.18242)
Supplement: Supplementary file 1 [file oncotarget-08-74096-s001.pdf]

Correlation analysis between molecular subtypes and Nottingham Prognostic Index in breast cancer

Supplementary Materials

Supplementary Table 1: Clinicopathological data for the entire patients cohorts

	Luminal A (*n* = 109)	Luminal B (*n* = 565)	HER2 overexpression (*n* = 85)	Basal-like (*n* = 117)	Unclassified (*n* = 166)
	n%	*n*%	*n*%	*n*%	*n*%
**NPI score**					
2.00-3.40	39 (35.8)	183 (32.4%)	16 (18.8%)	30 (25.6%)	44 (26.5%)
3.41-5.40	25 (22.9)	190 (33.6%)	27 (31.8%)	47 (40.2%)	37 (22.3%)
>5.40	3 (2.8%)	23 (4.1%)	5 (5.9%)	8 (6.8%)	6 (3.6%)
Absent	42 (38.5%)	169 (29.9%)	37 (43.5%)	32 (27.4%)	79 (47.6%)
**Grade**					
High histological grade	3 (2.8)	36 (6.4%)	9 (10.6%)	22 (18.8%)	6 (3.6%)
Intermediate histological grade	42 (38.5)	293 (51.9%)	31 (36.5%)	51 (43.6%)	61 (36.8%)
Low histological grade	17 (15.6)	50 (8.8%)	6 (7.0%)	6 (5.1%)	10 (6.0%)
Absent	47 (43.1)	186 (32.9%)	39 (45.9%)	38 (32.5%)	89 (53.6%)
**Tumor size**					
T1	39 (35.8%)	198 (35.0%)	29 (34.1%)	38 (32.5%)	47 (28.3%)
T2	37 (33.9%)	237 (41.9%)	27 (31.8%)	55 (47.0%)	64 (38.6%)
T3	5 (4.6%)	12 (2.1%)	6 (7.1%)	2 (1.7%)	7 (4.2%)
T4	6 (5.5%)	20 (3.6%)	3 (3.5%)	2 (1.7%)	5 (3.0%)
TX	22 (20.2%)	98 (17.3%)	20 (23.5%)	20 (17.1%)	43 (25.9%)
**Lymph nodes**					
N0	38 (34.9%)	220 (38.9%)	30 (35.3%)	51 (43.6%)	69 (41.5%)
N1	22 (20.2%)	152 (26.9%)	16 (18.8%)	28 (23.9%)	32 (19.3%)
N2	0 (0.0%)	2 (0.4%)	0 (0.0%)	0 (0.0%)	0 (0.0%)
NX	49 (45.0%)	191 (33.8%)	39 (45.9%)	38 (32.5%)	65 (39.2%)

Abbreviation: NR = not reach.

Supplementary Table 2: Clinicopathological data for the entire patients cohorts

	ER positive (*n* = 670)	ER negative (*n* = 216)	ER-NR (*n* = 156)	PR high expression (*n* = 456)	PR low expression (*n* = 105)	PR negative (*n* = 314)	PR-NR (*n* = 167)
	n%	***n%***	***n%***	***n%***	***n%***	***n%***	***n%***
**NPI score**							
2.00–3.40	221 (33.0%)	46 (21.2%)	45 (28.8%)	157 (34.3%)	33 (31.4%)	78 (24.8%)	44 (26.3%)
3.41–5.40	208 (31.0%)	80 (37.0%)	38 (24.5%)	138 (30.3%)	28 (26.7%)	122 (38.9%)	38 (22.8%)
> 5.40	25 (3.7%)	14 (6.5%)	6 (3.8%)	13 (2.9%)	11 (10.5%)	15 (4.8%)	6 (3.6%)
Absent	216 (32.3%)	76 (35.2%)	67 (42.9%)	148 (32.5%)	33 (31.4%)	99 (31.5%)	79 (47.3%)
**Grade**							
High histological grade	32 (4.8%)	39 (18.1%)	5 (3.2%)	18 (3.9%)	11 (10.5%)	41 (13.1%)	6 (3.6%)
Intermediate histological grade	332 (49.6%)	83 (38.3%)	63 (40.2%)	232 (50.9%)	47 (44.7%)	137 (43.6%)	62 (37.1%)
Low histological grade	68 (10.1%)	12 (5.6%)	9 (5.8%)	47 (10.3%)	11 (10.5%)	21 (6.7%)	10 (6.0%)
Absent	238 (35.5%)	82 (38.0%)	79 (50.6%)	159 (34.9%)	36 (34.3%)	115 (36.6%)	89 (53.3%)
**Tumor size**							
T1	238 (35.5%)	68 (31.5%)	45 (28.8%)	172 (37.7%)	31 (29.4%)	101 (32.2%)	47 (28.2%)
T2	269 (40.1%)	89 (41.2%)	62 (39.7%)	171 (37.5%)	51 (48.6%)	136 (43.3%)	62 (37.1%)
T3	16 (2.4%)	9 (4.2%)	7 (4.5%)	13 (2.9%)	1 (1.0%)	11 (3.5%)	7 (4.2%)
T4	26 (3.9%)	5 (2.3%)	5 (3.3%)	17 (3.7%)	5 (4.8%)	9 (2.8%)	5 (3.0%)
TX	121 (18.1%)	45 (20.8%)	37 (23.7%)	83 (18.2%)	17 (16.2%)	57 (18.2%)	46 (27.5%)
**Lymph nodes**							
N0	256 (38.2%)	85 (39.3%)	67 (42.9%)	169 (37.1%)	44 (41.9%)	126 (40.1%)	69 (41.3%)
N1	170 (25.4%)	47 (21.8%)	33 (21.2%)	114 (25.0%)	29 (27.6%)	74 (23.6%)	33 (19.8%)
N2	2 (0.3%)	0 (0.0%)	0 (0.0%)	0 (0.0%)	1 (1.0%)	1 (0.3%)	0 (0.0%)
NX	242 (36.1%)	84 (38.9%)	56 (35.9%)	173 (37.9%)	31 (29.5%)	113 (36.0%)	65 (38.9%)

Abbreviation: NR = not reach

Supplementary Table 3: Clinicopathological data for the entire patients cohorts

	HER2 positive (*n* = 196)	HER2 negative (*n* = 627)	HER2-NR (*n* = 219)	Ki67 high expression (*n* = 653)	Ki67 low expression (*n* = 170)	Ki67-NR (*n* = 219)
	***n*%**	***n*%**	***n*%**	***n*%**	***n*%**	***n*%**
**NPI score**						
2.00-3.40	60 (30.6%)	198 (31.6%)	54 (24.7%)	196 (30.0%)	61 (35.9%)	55 (25.1%)
3.41-5.40	66 (33.7%)	217 (34.6%)	43 (19.6%)	241 (36.9%)	38 (22.4%)	47 (21.5%)
>5.40	9 (4.6%)	30 (4.8%)	6 (2.7%)	33 (5.1%)	6 (3.5%)	6 (2.7%)
Absent	61 (31.1%)	182 (29.0%)	116 (53.0%)	183 (28.0%)	65 (38.2%)	111 (50.7%)
**Grade**						
High histological grade	13 (6.6%)	55 (8.8%)	8 (3.7%)	62 (9.5%)	5 (2.9%)	9 (4.1%)
Intermediate histological grade	110 (56.1%)	300 (47.8%)	68 (31.1%)	338 (51.8%)	66 (38.8%)	74 (33.8%)
Low histological grade	7 (3.6%)	67 (10.7%)	15 (6.8%)	49 (7.5%)	28 (16.5%)	12 (5.5%)
Absent	66 (33.7%)	205 (32.7%)	128 (58.4%)	204 (31.2%)	71 (41.8%)	124 (56.6%)
**Tumor size**						
T1	72 (36.7%)	217 (34.6%)	62 (28.3%)	223 (34.2%)	71 (41.8%)	57 (26.0%)
T2	74 (37.8%)	267 (42.6%)	79 (36.1%)	286 (43.8%)	58 (34.1%)	76 (34.7%)
T3	10 (5.1%)	15 (2.4%)	7 (3.2%)	19 (2.9%)	6 (3.5%)	7 (3.2%)
T4	4 (2.0%)	24 (3.8%)	8 (3.7%)	21 (3.2%)	7 (4.1%)	8 (3.7%)
TX	36 (18.4%)	104 (16.6%)	63 (28.7%)	104 (15.9%)	28 (16.5%)	71 (32.4%)
**Lymph nodes**						
N0	74 (37.8%)	252 (40.2%)	82 (37.4%)	266 (40.7%)	54 (31.8%)	88 (40.2%)
N1	49 (25.0%)	166 (26.5%)	35 (16.0%)	181 (27.7%)	33 (19.4%)	36 (16.4%)
N2	0 (0.0%)	2 (0.3%)	0 (0.00)	1 (0.2%)	1 (0.6%)	0 (0.0%)
NX	73 (37.2%)	207 (33.0%)	102 (46.6%)	205 (31.4%)	82 (48.2%)	95 (43.4%)

Abbreviation:NR = not reach
